# Surface runoff and soil erosion from *Nitisols* and *Ferralsols* as influenced by different soil organic carbon levels under simulated rainfall conditions

**DOI:** 10.1016/j.heliyon.2023.e17684

**Published:** 2023-06-30

**Authors:** Mercy K. Rugendo, Bernard M. Gichimu, Jayne N. Mugwe, Monicah Mucheru-Muna, Daniel N. Mugendi

**Affiliations:** a Department of Water and Agricultural Resource Management, University of Embu, P.O. Box 6-60100, Embu, Kenya; b Department of Agricultural Sciences and Technology, Kenyatta University, P.O. Box 43844-00100, Nairobi, Kenya; c Department of Environmental Sciences and Education, Kenyatta University, P.O. Box 43844-00100, Nairobi, Kenya

**Keywords:** Rainfall simulator, Sediment concentration, Surface runoff, Agricultural productivity, Soil organic carbon

## Abstract

Soil erosion poses a challenge to the environment and the sustainable use of natural resources, particularly in relation to agricultural production. The study aimed to assess the influence of different soil organic carbon (SOC) levels on runoff and soil erosion under varying levels of rainfall intensity. The study was conducted in pre-selected farmers' fields representing low, moderate and adequate SOC levels in *Nitisols* and *Ferralsols*. Two parallel experiments were set up in each type of soil using a split-plot layout arranged in Randomized Complete Block Design. The main plots were the different soil organic carbon levels while the sub-plots were the different simulated rainfall intensities. Rainfall simulation was then conducted to determine runoff and sediment losses on each soil type. The simulation was done using a land type sprinkler nozzle rainfall simulator (460 788 type) in an experimental plot of 1 m^2^, fenced with corrugated iron sheets with a small opening left for runoff collection. Runoff and sediment losses were determined from the volume collected in the jar. The data was subjected to analysis of variance and significant mean differences were determined using Tukey’s Honest Test at a 95% confidence level. Pearson correlation was applied to assess the relationship between runoff volume and sediment loss. The results showed that *Ferralsols* recorded significantly higher runoff and sediment losses compared to *Nitisols*, by 60.27% and 53.14% respectively. However, adequate SOC level portrayed a significant effect in reducing erosion in both soil types, where it reduced runoff and sediment loss by 45.30% and 48.38% in *Ferralsols* and by 65.31% and 48.22% in *Nitisols*, respectively. In both soil types, runoff yield was positively correlated to rainfall intensity while sediment yield was inversely correlated with SOC levels. Therefore, the study recommends incorporation of organic matter to adequate levels in both soils, for reduced soil erosion.

## 1 Introduction

Agriculture is the primary source of food for livelihoods in Sub-Saharan Africa (SSA) due to its vital role in dimensions that embody food security [1,2]. Despite this, the sector has been experiencing several challenges such as increasing soil erosion and land degradation which has subjected the region to a continued decline in per capita food availability [3,4]. The dynamics of population pressure on natural resources have exposed agriculture to agents of erosion [5]. As a result, approximately 33% of the soils in the world are already degraded and this could escalate to 90% by 2050 [6]. Lal et al. [7] further reported the status of most of the soil resources in the world, being fair, poor, or in very poor conditions and stresses that soil erosion is still a significant environmental and agricultural issue across the world. Therefore, there is a need to identify ways for targeting limited erosion.

Soil erosion poses the greatest threat to the productivity of arable lands globally [8,9]. Rapid nutrient depletion associated with soil erosion by runoff contributes to low agricultural productivity at the farm level [10,11]. According to Dohlman et al. [12], approximately 20% of cropland worldwide experiences low productivity due to soil degradation. Worldwide, it is estimated that soil erosion losses can lead up to 50% reduction in crop yields [13] and approximately 24 billion tonnes of fertile soil are lost in the world annually due to erosion [14]. Therefore, soil erosion by runoff remains a major concern especially in the developing countries where it seriously threatens agricultural productivity and food security [15]. This can significantly reduce the production capacity of agricultural lands [16]. For instance, the annual crop yield loss in Africa is estimated to be 280 million tons due to erosion [17]. In Kenya, it is feared that the current rate of soil degradation may hamper the projected 7% annual growth of the agricultural sector [18].

The annual rate of soil erosion caused by water is estimated to be between 20 and 30 gigatonnes on a global scale [19]. The rate translates to local averages and local peaks of 10–20 and 50–100 tonnes per hectare annually, respectively [20]. The substantial difference between the rates of soil erosion and soil formation, when taken into account over a human time scale, renders soil a non-renewable resource [21]. In the humid and semi-humid regions of Sub-Saharan Africa, the anticipated average yearly soil loss from water erosion is 50 tonnes per hectare [22]. In Kenya, 30% of the land is considered to be degraded, and 12 million people are thought to reside on the degraded land and every year, about 1.3 billion US dollars (USD) are spent on addressing land degradation [23]. The primary physical cause of land degradation is soil erosion estimated to be 72 tonnes per hectare yearly [24]. However, there is growing concern that climate change and/or enhanced climate variability will increase erosion, thus raising more concerns on how this will be reduced.

Previous studies have documented various methods of reducing soil erosion, including terracing, conservation tillage, cover crops, use of mulching, among others [25]. These methods aim to reduce soil loss by improving soil structure nd by increasing soil organic carbon [26]. Soil organic carbon (SOC) is the key component of soil organic matter (SOM) that plays a vital role in soil fertility, water-holding capacity, and soil stability [27]. Soil organic carbon has been shown to enhance soil stability and reduce soil erodibility, as well as increasing water infiltration and soil moisture retention, which can improve plant growth and further reduce erosion [28,29]. According to Olson et al. [30], SOC can affect soil erosion and runoff by influencing soil aggregate stability, soil water content, and soil infiltration rate. However, soil erosion is still evident in different types of soils and the optimal level of soil organic carbon that can reduce erosion has not been documented [31]. Therefore, investigating the level of SOC that can control erosion could provide a promising strategy for sustainable soil management.

Different amounts of organic matter in the soil have been reported to have an influence on soil characteristics, including bulk density, structure, water-holding capacity, and aggregate stability among others [[27], [28], [29]]. In fact, studies have shown that soils with weak structural development are more prone to erosion [32]. The situation becomes worse where the soils are highly weathered like it is the case with *Nitisols* and *Ferralsols* [33] which dominate the Central highlands of Kenya and which were evaluated in this study. Unfortunately, most of these soils are reported to have low organic matter and highly susceptible to soil erosion [[32], [33], [34]]. However, the amounts of organic matter vary depending on a number of variables, including, but not limited to, topography, climate, soil type, soil temperature, and soil moisture [35]. Most studies have focused on the impact of SOC on soil properties and processes and recommended soil management practices that could reduce soil erosion without considering the variability in SOC levels across different soils. Therefore, there is a need to explore the threshold value of organic carbon that can influence the reduction of soil erosion in *Nitisols* and *Ferralsols* [36].

The highly weathered soils in the Central highlands of Kenya usually receives high rainfall that is sometimes sporadic, short-lived and with intensive thunderstorms that are highly erosive [37,38]. These soils are therefore highly degraded thus requiring sustainable soil management strategies that can reduce the impact of soil erosion [15,39]. Although several studies have evaluated the impact of SOC on soil erosion and the relationship between SOC and its erosion rates, little is known about the optimal level of SOC that can effectively control erosion in *Nitisols* and *Ferralsols* as explained by Wang et al. [26]. This knowledge gap limits our ability to develop effective erosion control strategies that can promote SOC levels in *Nitisols* and *Ferralsols.* Soils containing organic matter have a better structure that improves water infiltration, and reduces the soil’s susceptibility to compaction, resulting to reduced erosion. Identifying such impactful SOC levels and their interaction with various soil types is key to decreasing the soil erosion caused by runoff. Therefore, this study sought to (1), determine the effects of different SOC levels in reducing surface runoff and soil erosion in the *Nitisols* and *Ferralsols* of Tharaka-Nithi County, Kenya.

## 2 Materials and methods

### 2.1 Description of the study area

**Fig. 1:**
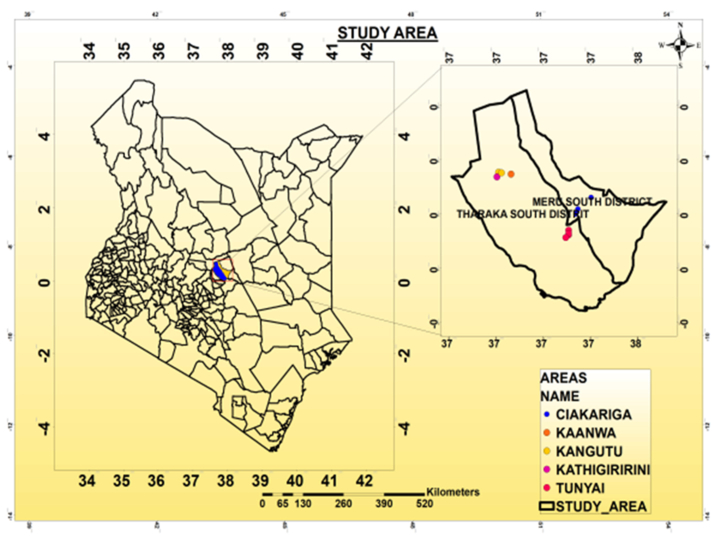
Map of the study area showing Meru-South and Tharaka-South Sub Counties.

The study was conducted in Meru South and Tharaka South Sub-Counties in Tharaka-Nithi County in Kenya (Fig. 1). On the first hand, Meru South Sub-County, the experimental site lies between latitude 00° 32′93″ N – 00° 32′40.1″ N and longitude 37° 67′19″ E − 37° 71′ 24.5″ E. The region lies in the Upper Midland (UM) 1 agro-ecological zone (AEZ) on the eastern slopes of Mount Kenya at an altitude of between 1100 and 1500 m above sea level (m.a.s.l.). The total annual rainfall is between 1200 and 1400 mm, and the annual average temperature is 20 °C. The rainfall pattern of the region is bimodal whereby, long rains fall from March to June and short rains from October to December. The soils are predominantly humic *Nitisols*, a typically deep and well-weathered soil with moderate to high inherent fertility [40]. The area is predominantly a maize and beans growing zone but coffee, tea and bananas are also grown.

On the other hand, Tharaka South Sub-County experiment was laid out between latitude 00° 18′ 09.5″ N – 00° 23′ 89.2″ N and longitude 37° 84′ 94.1″ E − 37° 87′ 51.4″ E. The region is situated between 700 and 900 m above sea level in the Lower Midland (LM) 5 agro-ecological zone on Mount Kenya’s eastern slopes [40]. The potential evapotranspiration rate is high and the mean annual temperature is 27 °C [40]. The average annual rainfall ranges from 500 to 750 mm [41] which is highly unpredictable and unevenly distributed [42]. There are two cropping seasons per year due to the bimodal rainfall pattern which includes long rains falling from mid-March to June and short rains from late October to December. The dominant soils are *Ferralsols,* which are highly weathered and have very low fertility due to their low mineral concentration [40]. The primary agricultural activity and a source of livelihood is livestock keeping [43]. The crops grown in Tharaka South Sub-County are predominantly sorghum, green grams, cowpeas, groundnut, maize, finger-millet and bulrush millet.

### 2.2 Conceptual framework

**Fig. 2:**
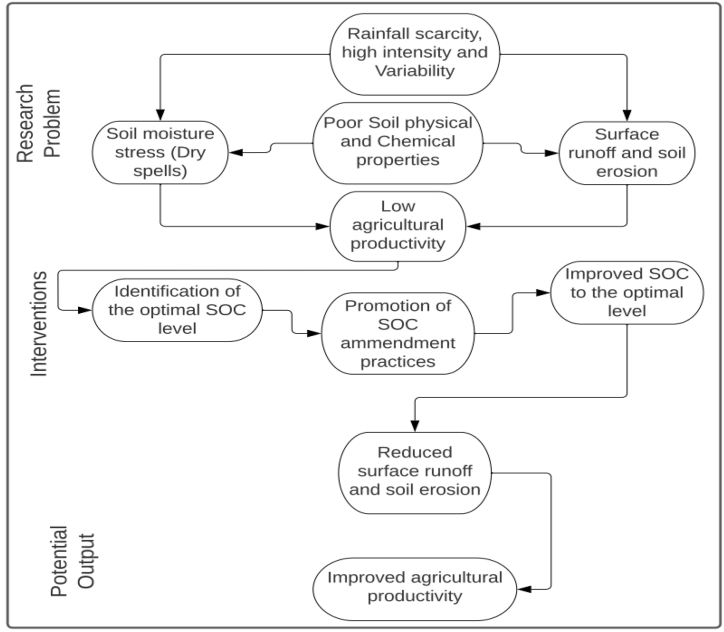
Conceptual framework showing the interrelation of the study variables.

Soil erosion, poor physical and chemical properties and soil moisture stress contribute to low agricultural productivity in Tharaka-Nithi County in Kenya [35]. Rainfall variability causes moisture stress to crops and contributes to soil erosion [37]. Reduced soil organic carbon results to reduced soil aggregate stability that renders soil highly susceptible to erosion and subsequent nutrient loss [30]. Identification of the SOC levels that can reduce soil erosion will be the basis of identifying soil management practices that would improve soil organic carbon to the optimal level, hence reduce surface runoff, improve soil moisture availability, and enhance agricultural productivity (Fig. 2).

### 2.3 Selection of experimental fields

A total of 57 farmers' fields (32 in Meru South and 28 in Tharaka South) were initially sampled purposively based on their existing agricultural activities. The main factors considered were; the existing soil and water management practices, type of fertilization used, type of crops grown in a span of 2 years, land preparation practices, use of irrigation, weeding practices and the crop yield in short and long rain seasons. A soil auger was used to collect soil samples from the sampled fields at depths that ranged from 0 to 15 cm using a soil auger and transported to the laboratory for SOC analysis. Subsequently, nine (9) fields were selected in each Sub-County (for each soil type) representing the three SOC levels (low, moderate and adequate) and replicated 3 times.

### 2.4 Experimental design and layout

An experimental set-up that allowed one to keep all the factors that influence runoff generation constant except soil organic carbon was used. Two parallel rainfall simulation experiments were set up in each of the two soils using a split-plot layout arranged in Randomized Complete Block Design (RCBD). The main plots were the different SOC levels i.e., low (1.0–1.5), moderate (1.5–2.5) and adequate (above 2.5) as recorded by Refs. [[44], [45], [46], [47], [48]]; while the sub-plots were different rainfall simulation intensities i.e., high (120 mm/h), moderate (100 mm/h) and low (80 mm/h). The variation in rainfall intensities was guided by the different rainfall amounts received annually in the two regions as estimated by Refs. [31,42]. At the start of the experiment, four rain gauges were used to measure simulated rainfall intensity (mm/hr) and at every start of the experiment, flow rate of the nozzles was calibrated to produce the three different rainfall intensities.

**Fig. 3:**
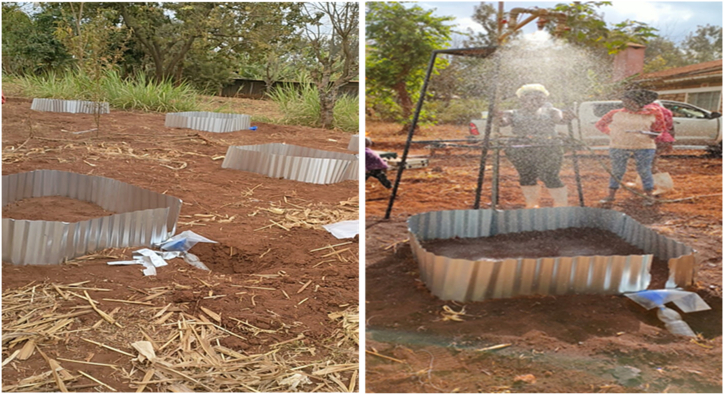
A partial view of the experimental set up displaying the plot layout (left) and the rainfall simulation (right) in Meru-South Sub County in Tharaka-Nithi County.

Rainfall simulations were carried out on fields during summer, before the short rains that fall from late October to December where there were no crops grown during that period [49,50]. In each soil type, a total of 27 rainfall simulations were done using a downward-oriented, single-nozzle, continuous-spray system [51]. The system consists of a land type sprinkler nozzle rainfall simulator, an axial-flow, wide-angle, full cone nozzle (460 788 type) designed to simulate rainfall at different rainfall intensities [52]. Pressure in the system was adjusted by a pressure regulator (NORGREN, type E2H- 4G: 0–100 kPa). The nozzle was positioned 2 m above the ground and directed to a small plot of 1 m^2^ plot that was fenced with corrugated iron sheets inserted 10 cm into the ground with 15 cm left above the surface to avoid overflow from the designated plot. Each plot, had a small outlet through which the runoff content was collected in a sampling jar for volume determination and analysis of the sediment yield. A polythene sheet was used to cover the funnel of the sampling jar to prevent direct rainfall from getting into the jar (Fig. 3).

### 2.5 Determination of surface runoff and soil erosion

In every test, the three rainfall intensities of 80, 100 and 120 mm/h were applied over a duration of 60 min and the annual rainfall simulated ranged from 1000 to 1500 mm. The runoff flow started at the point of soil saturation when the water started draining out from a slow drip to a continuous stream. The runoff samples were collected when the surface runoff started at the outlet of the plot. The runoff and sediment samples were continually collected into bottles and empty bottles were replaced every 1–3 min depending on the amounts of runoff and sediments produced. At rainfall termination, the runoff volumes collected from different experimental plots were packed and taken to the laboratory and measured using a graduated cylinder. The sediment samples were then filtered from the runoff water using Whatman filter papers and oven dried at 105 °C to constant dry weight and measured using an electronic scale used for weighing whose accuracy was 0.01 g. The total sediment loss per plot was calculated as shown in equation (1) (Okalebo et al., 2002).

(1) $$Total\;sediment\;loss\;\frac{kg}{ha}=\frac{sediment\;concentration\;\frac{g}{l}\times runoff\;volume\;\left(l\right)}{plot\;area\;\left(m^{2}\right)\times 10^{-1}}$$

### 2.6 Statistical analysis

The data was subjected to analysis of variance (ANOVA) using XLSTAT version 2022. Tukey’s Honest Significant Difference was used to separate the means at 95% level of confidence. Pearson correlation was done to establish the relationship between runoff and sediment loss.

## 3 Results

### 3.1 Impacts of soil type, organic carbon and rainfall intensities on runoff and soil erosion

**Fig. 4:**
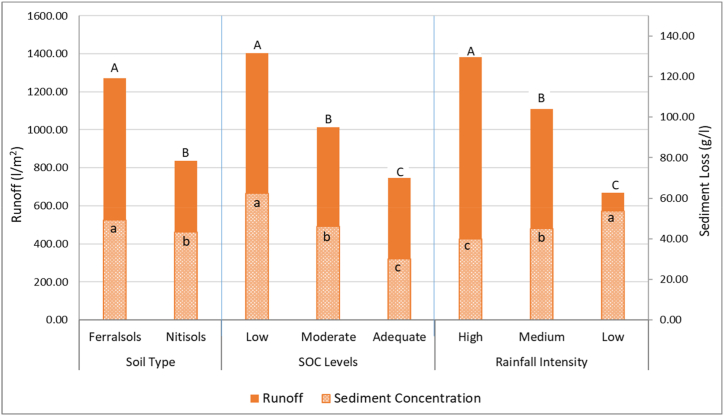
Runoff and sediment loss as affected by soil type, SOC levels and rainfall intensities in Tharaka South and Meru South Sub-Counties. *Means with different letters on the bar graph per group are significantly different at p < 0.05.*

Results of runoff and sediment loss as affected by soil type, soil organic carbon and rainfall intensities are displayed in Fig. 4. The three factors had a highly significant (p < 0.0001) effect on runoff and sediment loss. *Ferralsols* recorded significantly the highest runoff of 1270.93 l/m^2^ and also the highest sediment loss of 49.14 g/l compared to *Nitisols,* which recorded total run-off of 837.78 l/m^2^ and sediment loss of 43.32 g/l (Fig. 4). The runoff volume and sediment loss from soils with adequate organic carbon were significantly lower than those from soils with moderate organic carbon, which were also significantly lower than those from soils with low organic carbon (Fig. 4). On the other hand, the volume of runoff and loss of sediments under different rainfall intensities were found to be inversely related in that the runoff volume increased significantly with increasing rainfall intensities while the sediment loss decreased significantly with increasing rainfall intensities (Fig. 4). There were highly significant (p < 0.0001) interactions between soil type and SOC levels; soil type and rainfall intensity; and SOC levels and rainfall intensity for both runoff and sediment loss. This indicated that the different SOC levels and rainfall intensities had different effects on the different soil types.

### 3.2 Effects of soil organic carbon on runoff and soil erosion in *Ferralsols* and *Nitisols*

Soil organic carbon significantly influenced runoff volume in both *Ferralsols* and *Nitisols* (Table 1). The runoff volume and sediment loss were found to reduce significantly (p < 0.0001) as the soil organic carbon increased from low to adequate levels but there was no significant difference in runoff volumes recorded under *Nitisols* with low and moderate SOC levels. *Ferralsols* had a 54.4% higher runoff volume (1703.33 l/m^2^) compared to *Nitisols* (1103.33 l/m^2^) under low SOC level. Regarding sediment loss, *Ferralsols* with low SOC levels recorded a 16.2% higher value (67.25 kg/ha) compared to *Nitisols* (57.70 kg/ha). For moderate SOC levels, *Ferralsols* had a 7.7% higher sediment loss (47.62 kg/ha) than *Nitisols* (44.44 kg/ha). A comparison of the two soils showed that *Ferralsols* recorded significantly (p < 0.01) higher runoff volumes and sediment losses than *Nitisols* at all three levels of soil organic carbon (Table 1).

**Table 1:** Effects of SOC on runoff and sediment loss in both soil types.

Variable	SOC Levels	*Ferralsols*	*Nitisols*	SE	P Value
Runoff (l/m^2^)	Low	1703.33^a A^	1103.33^a B^	46.91	<0.0001
	Moderate	1337.78^b A^	689.44^b B^	26.38	<0.0001
	Adequate	771.67^c A^	720.56^b B^	9.49	0.003
	SE	30.92	20.56		
	P Value	<0.0001	<0.0001		
Sediment loss (kg/ha)	Low	67.25^a A^	57.70^a B^	0.83	<0.0001
	Moderate	47.62^b A^	44.44^b B^	0.67	0.008
	Adequate	32.54^c A^	27.82^c B^	0.94	0.005
	SE	0.95	0.80		
	P Value	<0.0001	<0.0001		

**Legend:** SOC – Soil Organic Carbon; SE - Standard Error. Means with different small letters within the column and different capital letters within the row are significantly different at p < 0.05.

### 3.3 Effects of rainfall intensity on runoff and soil erosion in *Ferralsols* and *Nitisols*

Varying rainfall intensities were found to have a highly significant (p < 0.0001) influence on both runoff volume and sediment loss in both *Ferralsols* and *Nitisols* (Table 2). However, the effect of rainfall intensity on runoff volume was inversely related to that of sediment loss in that the runoff volume increased significantly with the increase in rainfall intensities while the sediment loss decreased significantly with the increase in rainfall intensities. Under a rainfall intensity of 120 mm/h, *Ferralsols* had a 61.5% higher runoff volume (1707.78 l/m^2^) compared to *Nitisols* (1058.89 l/m^2^). At a rainfall intensity of 100 mm/h, *Ferralsols* had a 55.3% higher runoff volume (1351.67 l/m^2^) than *Nitisols* (870.00 l/m^2^). Regarding sediment loss, at a rainfall intensity of 120 mm/h, *Ferralsols* experienced a 29.9% higher sediment loss (45.14 kg/ha) compared to *Nitisols* (34.92 kg/ha). Under a rainfall intensity of 100 mm/h, *Ferralsols* had a 13.6% higher sediment loss (47.98 kg/ha) compared to *Nitisols* (42.21 kg/ha). However, at a rainfall intensity of 80 mm/h, there was no significant difference in sediment loss between *Ferralsols* (54.29 kg/ha) and *Nitisols* (52.83 kg/ha). A comparison of the two soils showed that *Ferralsols* recorded significantly (p < 0.01) higher runoff volumes and sediment loss than *Nitisols* at all three rainfall intensities except for the sediment loss at the lowest rainfall intensity (80 mm/h) where the two soils were not significantly different (Table 2).

**Table 2:** Effects of rainfall intensity on runoff and sediment loss in both soil types.

Variable	Rainfall Intensity	*Ferralsols*	*Nitisols*	SE	P Value
Run-off (lm^2^)	120 mm/h	1707.78^a A^	1058.89^a B^	39.71	<0.0001
	100 mm/h	1351.67^b A^	870.00^b B^	33.43	<0.0001
	80 mm/h	753.33^c A^	584.44^c B^	11.41	<0.0001
	SE	30.92	20.56		
	P Value	<0.0001	<0.0001		
Sediment Loss (kg/ha)	120 mm/h	45.14^b A^	34.92^c B^	0.82	<0.0001
	100 mm/h	47.98^b A^	42.21^b B^	1.08	0.004
	80 mm/h	54.29^a A^	52.83^a A^	1.72	0.178^NS^
	SE	0.95	0.80		
	P Value	<0.0001	<0.0001		

**Legend:** SE - Standard Error. Means denoted by different small letters within the same column and different capital letters within the same row are significantly different at p < 0.05.

### 3.4 Interaction between the soil organic carbon and rainfall intensity on runoff and soil erosion

There was a highly significant interaction (p < 0.0001) between soil organic carbon and rainfall intensity on runoff and sediment loss (Table 3). In both soils, the highest (p < 0.05) runoff volumes were observed in a combination of low SOC levels and high rainfall intensity while the lowest runoff volumes were obtained under adequate SOC levels and lowest rainfall intensity. Under the low SOC and rainfall treatment, *Ferralsols* had a runoff volume that was approximately 11.1% higher (900.00 l/m^2^) compared to *Nitisols* (810.00 l/m^2^). In the low SOC and medium rainfall treatment, *Ferralsols* had a runoff volume that was approximately 61.2% higher (1826.67 l/m^2^) than *Nitisols* (1133.33 l/m^2^). For the low SOC and high rainfall treatment, *Ferralsols* had the highest runoff volume, which was approximately 74.7% higher (2383.33 l/m^2^) than *Nitisols* (1366.67 l/m^2^). Regarding sediment loss, under the low SOC and rainfall treatment, *Ferralsols* recorded a sediment loss that was approximately 3.3% higher (73.60 kg/ha) compared to *Nitisols* (71.19 kg/ha). For the low SOC and high rainfall treatment, *Ferralsols* had a sediment loss that was approximately 37.1% higher (63.28 kg/ha) than *Nitisols* (46.14 kg/ha). Apparently, the combined influence of SOC levels and rainfall intensity on runoff followed a near-consistent trend in most of the treatments. This was an indication that runoff volumes increase with an increase in rainfall intensity and more runoff is experienced in soils with low organic carbon levels as compared to those with adequate or high organic carbon levels (Table 3). In both soils, the scenario was somehow different for the sediment loss whereby, high organic carbon levels in the soil were found to significantly reduce the sediment loss while higher sediment loss was observed under low rainfall than under high rainfall intensities. Consequently, the highest (p < 0.05) sediment loss was observed in a combination of low SOC levels and low rainfall intensity while the lowest sediment loss occurred under adequate SOC levels and highest rainfall intensity. A consistent trend was observed in both soils whereby the sediment loss reduced with increasing SOC levels but for any given SOC levels, the sediment loss reduced with increasing rainfall intensity (Table 3).

**Table 3:** Combined treatment effects on runoff and sediment loss.

Variable	Treatment	*Ferralsols*	*Nitisols*
Runoff	Low SOC*Low Rainfall	900.00^d^	810.00^cd^
	Low SOC*Medium Rainfall	1826.67^b^	1133.33^b^
	Low SOC*High Rainfall	2383.33^a^	1366.67^a^
	Moderate SOC*Low Rainfall	770.00^de^	473.33^e^
	Moderate SOC*Medium Rainfall	1453.33^c^	710.00^d^
	Moderate SOC*High Rainfall	1790.00^b^	885.00^cd^
	Adequate SOC*Low Rainfall	590.00^e^	470.00^e^
	Adequate SOC*Medium Rainfall	775.00^de^	766.67^cd^
	Adequate SOC*High Rainfall	950.00^d^	925.00^c^
	Standard Error	53.56	35.61
	P Value	<0.0001	<0.0001
Sediment Loss (Kg/ha)	Low SOC*Low Rainfall	73.60^a^	71.19^a^
	Low SOC*Medium Rainfall	64.87^b^	55.77^b^
	Low SOC*High Rainfall	63.28^b^	46.14^c^
	Moderate SOC*Low Rainfall	53.49^c^	53.92^b^
	Moderate SOC*Medium Rainfall	46.24^cd^	44.09^c^
	Moderate SOC*High Rainfall	43.15^de^	35.32^d^
	Adequate SOC*Low Rainfall	35.79^ef^	33.38^de^
	Adequate SOC*Medium Rainfall	32.83^f^	26.79^ef^
	Adequate SOC*High Rainfall	29.01^f^	23.31^f^
	Standard Error	1.64	1.38
	P Value	<0.0001	<0.0001

Legend: Means in the column with different letters are significantly different at p < 0.05.

### 3.5 Relationship between runoff and sediment loss

Pearson correlation was carried out to evaluate the relationship between runoff and sediment loss at different factor combinations as shown in Table 4. There was a significant (p < 0.05) positive correlation between runoff and sediment loss in *Ferralsols,* indicating that the sediment loss would generally increase as the runoff volume increased. Further analysis showed that there were significant (p < 0.05) negative correlations between runoff and sediment loss at moderate and low SOC levels but there was no significant correlation between the two variables under adequate SOC levels. However, there were significant (p < 0.05) positive correlations between runoff and sediment levels at different rainfall intensities in *Ferralsols* (Table 4). However, for the interactions between moderate SOC levels and all rainfall intensities combined, the coefficients were significantly negative (r = −0.968, p < 0.0001; low SOC level and all combined rainfall intensities: r = −0.898, p < 0.001). For *Nitisols,* a combined analysis indicated that there was no correlation between the two variables indicating that the two were differently influenced by different combinations of SOC levels and rainfall intensities. The two variables were significantly (p < 0.05) negatively correlated at different SOC levels and significantly (p < 0.05) positively correlated at high and low rainfall intensities. However, there was no significant correlation between the two variables at moderate rainfall intensity. When both soil types were analyzed with factor interactions, a significant (p < 0.05) positive correlation was noted between runoff and sediment loss indicating that an increase in runoff would generally result in higher sediment loss. There was no significant correlation between runoff and sediment loss in both soils under moderate and low SOC levels with rainfall intensity held constant but the two variables were found to be significantly (p < 0.05) inversely correlated under adequate SOC levels. On the other hand, with SOC levels held constant, the results showed highly significant (p < 0.0001) positive correlations between runoff and sediment loss (Table 4).

**Table 4:** Relationship between runoff and sediment loss in both soil types.

Soil Type	SOC Levels	Rainfall Levels	Coefficient	P Value
*Ferralsols*	Adequate	All Combined	−0.567^NS^	0.111
*Ferralsols*	Moderate	All Combined	−0.968***	<0.0001
*Ferralsols*	Low	All Combined	−0.898***	<0.001
*Ferralsols*	All Combined	High (120 mm/h)	0.943***	<0.0001
*Ferralsols*	All Combined	Medium (100 mm/h)	0.932***	<0.0001
*Ferralsols*	All Combined	Low (80 mm/h)	0.967***	<0.0001
*Ferralsols*	All Combined	All Combined	0.418*	0.030
*Nitisols*	Adequate	All Combined	−0.915***	0.001
*Nitisols*	Moderate	All Combined	−0.931***	<0.0001
*Nitisols*	Low	All Combined	−0.921***	<0.0001
*Nitisols*	All Combined	High (120 mm/h)	0.789*	0.011
*Nitisols*	All Combined	Medium (100 mm/h)	0.646^NS^	0.060
*Nitisols*	All Combined	Low (80 mm/h)	0.839**	0.005
*Nitisols*	All Combined	All Combined	0.088^NS^	0.661
Both Combined	Adequate	All Combined	−0.590*	0.010
Both Combined	Moderate	All Combined	−0.340^NS^	0.167
Both Combined	Low	All Combined	−0.214^NS^	0.393
Both Combined	All Combined	High (120 mm/h)	0.891***	<0.0001
Both Combined	All Combined	Medium (100 mm/h)	0.774***	<0.0001
Both Combined	All Combined	Low (80 mm/h)	0.794***	<0.0001
Both Combined	All Combined	All Combined	0.346*	0.010

Legend: NS – Not significant; *Significant at 5%; **Significant at 1%; ***Significant at 0.1%.

**Fig. 5:**
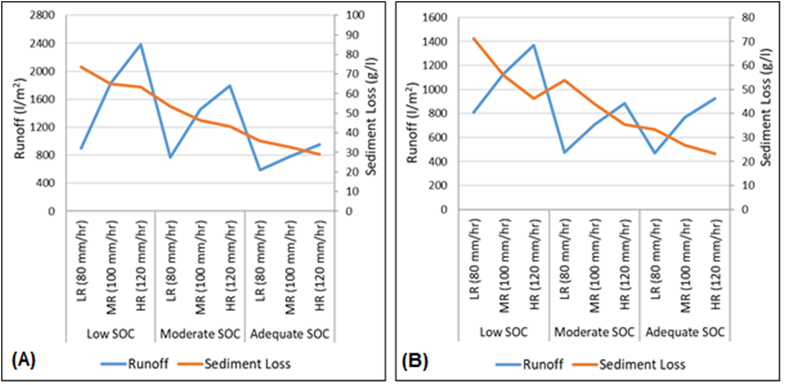
Relationship between runoff and sediment loss against varying levels of soil organic carbon (SOC) and rainfall intensity in *Ferralsols* (A) and *Nitisols* (B) (Tharaka-South and Meru-South Sub-Counties). LR – Low Rainfall; MR – Medium Rainfall; HR – High Rainfall.

The general effect of soil organic carbon levels and rainfall intensity on runoff volume and sediment loss on both soils appeared to follow a similar trend (Fig. 5). The two variables were found to decrease with an increase in soil organic carbon but changes in rainfall intensity resulted in a discordant effect on runoff and sedimentation. Apparently, the increase in rainfall intensity appeared to have a strong effect on the runoff volume increasing it beyond the arresting ability of the available soil organic carbon levels. On the contrary, the soil organic carbon levels in both soils appeared to effectively modify the levels of sedimentation to somewhat more stabilized levels regardless of the rainfall intensity (Fig. 5).

## 4 Discussion

The results obtained from this study showed that *Ferralsols* recorded significantly higher runoff and sediment loss compared to *Nitisols* since the latter have deep and stable structure with favorable physical properties compared to the former [53]. Similar observation was made by Abah and Petja [54], who found *Ferralsols* to have a weaker, very fine, medium, and coarse subangular blocky, and porous massive structure than *Nitisols,* which have a moderate to strong, fine to coarse, angular, and subangular blocky structure. This finding corroborates with the findings of Ngunjiri et al. [55] who found that runoff is more pronounced in *Ferralsols* as compared to humic *Nitisols* due to their soil compaction and crusting characteristic. Dotto et al. [56] also reported that *Ferralsols* are more weathered soils due to their high levels of sand content which makes the soil more prone to erosion. There was a high incidence of runoff in *Ferralsols* as compared to *Nitisols* even under low rainfall intensities. The properties of the soil type in combination with the climatic impacts in the area explains the high runoff volume observed in *Ferralsols* despite the low rainfall intensities [57,58]. The findings of this study agree with Ngetich et al. [59] who reported that surface seals and crusts occur in marginal areas with primarily silty soils, high rainfall intensities, and frequent dry periods, altering the hydrological parameters of a soil. Soil crusting could also be an indication of high runoff generation as reported by Cheng et al. [60]. Furthermore, Rockström et al. [61] found that crop fields can produce a large amount of surface over-land flow when rainfall intensities are combined with soil types that are vulnerable to crust formation.

The sediment loss and runoff volume from soils with adequate soil organic carbon were significantly lower than those from soils with moderate and low organic carbon due to the potential of the organic carbon in improving the soil aggregate stability and structure [62,63]. This finding agrees with Panagos et al. [64] and Krull et al. [65] who reported that adequate soil organic matter improves the soil's physical attributes such as soil structure which increases water infiltration rate and consequently reduces runoff and sediment loss. Saha et al. [66] sought to describe the relationship between soil physical attributes and soil quality and noted that soil organic matter directly affected the soil physical properties and microbial biomass hence adequate soil organic matter would primarily result to improved soil physical health and thus reduced runoff and sediment loss. Further reports indicate that higher levels of soil organic matter have a significant effect on soil bulk density [67], soil aggregation [68,69], soil structure [70], soil moisture-retention capacity [71] and infiltration rate [72], thus resulting in increased resistance to soil erosion, as corroborated in this study.

Apart from the SOC levels, the rainfall intensity had a substantial impact on both runoff volume and sediment loss. Similar findings were reported by Zhao et al. [73] who found that different rainfall regimes affect runoff and soil erosion differently. The highest rainfall intensity of 120 mm/h was found to cause significantly more runoff compared to the medium (100 mm/h) and low (80 mm/h) rainfall intensities. This was an indication that as rainfall intensity increased, the volume of water falling on the soil surpassed the soil infiltration rate thus resulting in runoff. In other words, runoff is produced when the rainfall intensity exceeds the soil’s capacity for water infiltration. The results of this study concur with the earlier findings by Ngetich et al. [74], Zhao et al. [75] and Zhang et al. [76] who found the rainfall intensity to be the major determining factor in the observed variability in surface runoff. Similar results were also reported in several other related studies [[77], [78], [79]].

Unlike runoff which was directly proportional to the rainfall intensity, an inverse relationship was observed between the rainfall intensity and sediment loss. Therefore, the sediment losses were found to decrease significantly as the rainfall intensity increased. This finding contradicted several earlier reports that higher rainfall intensities cause relatively sediment losses due to the higher kinetic energy and impact of their raindrops on soil particle detachment [[80], [81], [82], [83]]. The inverse relationship that was observed in this study between the rainfall intensity and sediment losses may have emanated from soil sealing which may have occurred after swelling of soil particles thus reducing the rate of infiltration which resulted in the increased runoff but reduced sediment losses. A similar hypothesis was drawn by Duan et al. [84]. Failure of sediment loss to increase proportionally with runoff may also be attributed to low slope gradient of the experimental plots. According to Zhang et al. [85], the slope gradient has a significant effect on the soil’s ability to detach and that soil detachment is much lower at a slope gradient of 0°. The authors further explained that the detached soil at near flat gradient forms a depositional screen that limits maximal raindrop penetration which results in the increased runoff but prevents continued erosion. In other words, minimal or no slope converts the erosion process from a detachment-limiting regime to a sediment transport-limiting regime. The findings are also consistent with those of Van Dijk et al. [86] who indicated that subsurface flows are far more likely to be facilitated by a prolonged, low-intensity rainfall pattern than a short, high-intensity rainfall event.

There was a significant interaction between soil organic carbon and rainfall intensities on runoff and sediment loss in both soils. The interaction showed that although high rainfall intensity was associated with high runoff, the level of organic carbon in the soil played a significant intervening role in modifying the effect of runoff. This confirmed the positive effect of soil organic matter in reducing runoff through improving soil infiltration and retention capacity [87]. The observation corroborates findings by Kiboi et al. [88], Bolo et al. [89] and Nyamwange et al. [90] that enhancement of the organic carbon in the soil reduces the exposure of the soil and improves microbial activity which results in improved soil structure. High soil organic carbon levels also appeared to modify the sediment loss at different rainfall intensities. González et al. [91] also noted that although the amount, size, and major components of the additional organic matter determine how soil characteristics and soil loss are affected by organic matter, the resulting effect is improved soil physical qualities that ultimately reduce sediment loss. Water-induced soil erosion is a selective process that typically eliminates soil elements with the smallest size and lowest density [92]. Consequently, the sediments are often enriched with fine silt and clay-sized particles that contain the most stable forms of soil organic carbon in the soil [93,94]. Therefore, this study’s findings suggest that adequate organic matter in the soil significantly results a reduction in runoff volume and sediment loss and also a decrease of carbon leaching into sediments; so, it also contributes to soil sequestration.

The relationship between runoff volume and sediment loss showed that if all the factors were to be held constant, the runoff volume would be directly proportional to sediment loss. This observation was due to the transportation of soil particles that mainly occurs through overland flow. However, when the two different soils were considered separately, the results still showed a direct relationship between runoff and sediment loss in *Ferralsols* but the two variables portrayed no significant relationship between them in *Nitisols*. This may be attributed to the fact that runoff volume is identified as a significant variable for sediment yield [[95], [96], [97], [98], [99]]. The findings of this study also agree with that of Endale et al. [100] who pointed out that, the likely explanation regarding no significant relationship of the variables in *Nitisols* could be due to their soil conditions where they are less compacted. *Ferralsols* showed high runoff volume and sediment yield, which would most probably be due to their high silt and extremely fine sand concentration and corresponding lower coarse fragment composition [96,97]. These findings contradict the commonly reported results by Liu et al. [101] and Yan et al. [102] which indicate a significant increase in runoff and in sediment yields following high rainfall intensities as influenced by slope gradient.

Correlation between runoff and sediment loss at varying levels of soil organic carbon but with rainfall intensity held constant showed either lack of or inverse relationship between the two variables, which was more evident in *Ferralsols* than in *Nitisols*. This indicated that although the organic matter level in the soil plays a major role in preventing soil erosion, the type of soil also has an important influence on erosion. This observation is in line with that of Fufa et al. [103] and Ma et al. [104] who concluded that the prediction of erodibility based on measured soil properties could be significantly influenced by its rock fragment content. This finding may be explained by *Ferralsols*' high rock-fragment content and low organic matter content, both of which had confounding effects on the generation of runoff and the subsequent sediment yield. On the other hand, the runoff volume was found to be directly proportional to the sediment loss at different rainfall intensities when the level of soil organic carbon was held constant. This was a further indication that runoff is the major channel of sediment loss and therefore reducing runoff is the best mitigating strategy for reducing sediment loss in a given scenario. This agrees with Solgi et al. [105] and Mandal et al. [106] who found that runoff can be used as an indicator of measuring soil erosion.

## 5 Conclusion

This study assessed the resilience of *Nitisols* and *Ferralsols* to runoff and sediment loss as influenced by different soil organic carbon levels and different rainfall intensities. The study established that adequate levels of soil organic carbon in both soil types have a significant influence in reducing runoff and sediment loss under different rainfall intensities. Thus, improvement of SOC to adequate levels through soil management practices that can improve soil organic matter should be considered to minimize soil erosion, particularly in regions that are prone to high rainfall intensities. The farmers in such regions should be encouraged to adopt soil management practices that would assist in improving the soil organic matter for sustainable agricultural production. The study therefore provides valuable information to farmers, policymakers, and researchers on the importance of soil organic matter in soil conservation. However, considering the variable profile of rainfall intensity in artificial rainfall events, further investigation of runoff and soil erosion under natural rainfall intensities is merited. In addition, future research could focus on identifying the most effective soil management practices for enhancing soil organic carbon levels in *Nitisols* and *Ferralsols.*

## Author contribution statement

Mercy Rugendo: Conceived and designed the experiments; Performed the experiments; Analyzed and interpreted the data; Wrote the paper.

Bernard M. Gichimu; Jayne N. Mugwe; Monicah Mucheru-Muna: Conceived and designed the experiments; Analyzed and interpreted the data; Wrote the paper.

Daniel N. Mugendi: Conceived and designed the experiments; Contributed reagents, materials, analysis tools or data; Wrote the paper.

## Funding statement

This work was supported by the Flemish Interuniversity Council-University Development Co-operation through the VLIR-UOS Project on *“Climate-Smart Options Allowing Agricultural Intensification among Smallholder Farmers in the Dry Zones of the Central Highlands of Kenya”.*

## Data availability statement

The original data of this paper were mainly obtained through the authors' experiment and will be made available on request.

## Declaration of competing interest

The authors declare that they have no known competing financial interests or personal relationships that could have appeared to influence the work reported in this paper
